# Heart Rate Variability Modulates Interoceptive Accuracy

**DOI:** 10.3389/fnins.2020.612445

**Published:** 2021-01-11

**Authors:** Alexander Lischke, Rike Pahnke, Anett Mau-Moeller, Matthias Weippert

**Affiliations:** ^1^Department of Psychology, University of Greifswald, Greifswald, Germany; ^2^Department of Sport Science, University of Rostock, Rostock, Germany

**Keywords:** heartbeat detection, heart rate variability, interoception, attention, vagal tone, emotion

## Abstract

Our emotional experiences depend on our interoceptive ability to perceive and interpret changes in our autonomous nervous system. An inaccurate perception and interpretation of autonomic changes impairs our ability to understand and regulate our emotional reactions. Impairments in emotion understanding and emotion regulation increase our risk for mental disorders, indicating that interoceptive deficits play an important role in the etiology and pathogenesis of mental disorders. We, thus, need measures to identify those of us whose interoceptive deficits impair their emotion understanding and emotion regulation. Here, we used cardiac measures to investigate how our ability to engage prefrontal and (para-)limbic brain region regions affects our ability to perceive and interpret cardiac changes. We administered a heartbeat detection task to a sample of healthy individuals (*n* = 113) whose prefrontal-(para-) limbic engagement had been determined on basis of a heart rate variability recording. We found a positive association between heartbeat detection and heart rate variability, implying that individuals with higher heart rate variability were more accurate in heartbeat detection than individuals with lower heart rate variability. These findings suggest that our interoceptive accuracy depends on our prefrontal-(para-)limbic engagement during the perception and interpretation of cardiac changes. Our findings also show that cardiac measures may be useful to investigate the association between interoceptive accuracy and prefrontal-(para-)limbic engagement in a time- and cost-efficient manner.

## Introduction

Interoception refers to our ability to perceive and interpret changes in our autonomous nervous system (Craig, 2002). Cardiac and respiratory activity are examples of autonomic processes that frequently change during emotional events (Kreibig, 2010). The perception and interpretation of these autonomic changes forms the basis of our emotional experiences and helps us to understand and to regulate our emotional reactions (Critchley and Garfinkel, 2017). For instance, those of us who are sensitive to cardiac changes have less difficulties in understanding and responding to emotional events than those of us who are insensitive to cardiac changes (Fustos et al., 2013; Shah et al., 2017; Lischke et al., 2020a, b). Given that emotion understanding and emotion regulation is central for our mental health (Gross and Jazaieri, 2014), it is not surprising that alterations in the perception and interpretation of autonomic changes increase our risk for mental disorders (Paulus and Stein, 2010). For instance, those of us whose cardiac sensitivity is in the abnormal range have more mental health problems than those of us whose cardiac sensitivity is in the normal range (Pollatos et al., 2007b, 2009; Herbert et al., 2011; Shah et al., 2016). The way we perceive and interpret autonomic changes, thus, appears to be of utmost importance for our mental health.

To understand how we perceive and interpret autonomic changes, we need measures that differentiate between different aspects of interoception (Critchley and Garfinkel, 2017). Fortunately, these measures have already been developed by researchers. Depending on the research question, these measures assess interoceptive accuracy (i.e., objective accounts of interoception), interoceptive sensibility (i.e., subjective accounts of interoception) or interoceptive awareness (i.e., correspondence between objective and subjective accounts of interoception). The most popular measure is the heartbeat detection task (Schandry, 1981), an interceptive accuracy task that requires the tracking of heartbeats within different time intervals. By employing the heartbeat detection task, researchers were able to identify a network of brain regions that is relevant for the perception and interpretation of cardiac changes (Schulz, 2016). This network comprises several brain regions but prefrontal and (para-)limbic brain regions appear to be the most important ones. Prefrontal and (para-)limbic show the most pronounced activity changes during the perception and interpretation of cardiac changes (Critchley et al., 2004; Pollatos et al., 2005; Kuehn et al., 2016), implying a close association between prefrontal-(para-)limbic activity and interoceptive accuracy. It may, thus, be possible that our ability to perceive and interpret cardiac changes depends on our ability to engage prefrontal and (para-)limbic brain regions for this matter.

To test this possibility, we administered measures of interoceptive accuracy and prefrontal-(para-)limbic engagement to a sample of young adults. Interoceptive accuracy was measured with the heartbeat detection task and prefrontal-(para-)limbic engagement was measured with a heart rate recording. The heart rate recording was used for the determination of parasympathically induced heart rate changes, a measure of vagally mediated heart rate variability (Shaffer and Ginsberg, 2017). Parasympathetically induced heart rate changes are closely associated with activity changes in prefrontal and (para-)limbic brain regions (Thayer et al., 2012; Ruiz Vargas et al., 2016), indicating that vagally mediated heart rate variability reflects prefrontal-(para-)limibic engagement (Thayer and Lane, 2009; Smith et al., 2017). The heart rate recording, thus, allowed us to investigate the association between interoceptive accuracy and prefrontal-(para-)limbic engagement in an unobtrusive manner. In light of previous findings showing that activity changes in prefrontal and (para-)limbic brain regions are positively associated with the perception and interpretation of cardiac changes (Critchley et al., 2004; Pollatos et al., 2005; Kuehn et al., 2016), we expected to find a similar association between vagally mediated heart rate variability and heartbeat detection. Preliminary findings suggest that vagally mediated heart rate variability may be positively associated with heartbeat detection (Owens et al., 2018). We, thus, expected to find a positive rather than negative association between vagally mediated heartrate variability and heartbeat detection.

## Materials and Methods

### Participants

We based our participant recruitment on an *a priori* power analysis with G^∗^Power 3.1.9.2 (Faul et al., 2009). The power analysis suggested that 82 participants would provide sufficient data to detect meaningful associations between vagally mediated heartrate variability and heartbeat detection in our analyses [correlation analysis (two-tailed): α = 0.05, 1-*ß* = 0.80, *r* = 0.30; regression analyses (total number of predictors: 8, number of tested predictors: 1): α = 0.05, 1-*ß* = 0.80, *f*^2^ = 0.15]. Following this suggestion, we recruited 113 participants for the study (see Table 1). In order to be included in the study, the participants had to be native speakers and to be aged between 18 and 35 years. Participants who were currently in psychotherapeutic treatment were excluded from the study. Inclusion and exclusion of participants was determined on basis of an in-house questionnaire assessing sociodemographic (age and sex), anthropometric (height and weight) and medical (physical activity in terms of aerobic fitness, smoking status, medication status, treatment status) information (Lischke et al., 2018a). All participants that were included in the study provided written-informed consent to the study protocol. The study protocol, which had been approved by the local ethics committee, was carried out in accordance with the Declaration of Helsinki.

**TABLE 1:** Participant characteristics.

	M (SD)	95% CI
Sex (f/m, n)	40/69	
Age	26.30 (3.91)	[25.55, 27.04]
Tobacco use (n)	28	
Medication use (n)	10	
Anti-allergic medication	3	
Endocrine medication	5	
Psychotropic medication	2	
Contraceptive use (n)	22	
Unspecified contraceptives	19	
Androgenic contraceptives	1	
Anti-androgenic contraceptives	2	
Body mass index (kg/m^2^)	22.90 (2.83)	[22.40, 23.42]
Physical activity (h/week)	6.19 (3.53)	[5.57, 6.86]
Respiratory activity (log pHF, Hz)	−0.71 (0.10)	[−0.72, −0.69]
Heart rate (bpm)	74.70 (12.20)	[72.25, 76.88]
Heart rate variability		
RMSSD (ms)	43.54 (26.19)	[38.51, 48.79]
log RMSSD (ms)	1.57 (0.25)	[1.52, 1.62]
pNN50 (%)	20.72 (17.66)	[17.35, 24.20]
log pNN50 (%)	1.14 (0.48)	[1.04, 1.23]
Heartbeat detection		
HBD_SC_	0.69 (0.18)	[0.66, 0.72]
HBD_GA_	0.60 (0.26)	[0.56, 0.65]

*pHF, peak of high frequency band (Shaffer and Ginsberg, 2017); RMSSD, root mean square of successive differences between consecutive heartbeats (Shaffer and Ginsberg, 2017); pNN50, number of successive heartbeat interval pairs that differ more than 50 ms divided by the total number of all heartbeat intervals (Shaffer and Ginsberg, 2017); HBD_SC_, Heartbeat detection – traditional index (Schandry, 1981); HBD_GA_, Heartbeat detection – alternative index (Garfinkel et al., 2015).*

### Procedure

We used a heart rate recording to determine participants’ vagally mediated heart rate variability and heartbeat detection. Each recording session was scheduled during the daytime (at least 2 h after wakening time and 5 h before sleeping time) to control for circadian and diurnal variations in participants’ heart rate (Yamasaki et al., 1996; Bonnemeier et al., 2003). Before we started with the recording session, we asked the participants to use the bathroom. This allowed us to rule out that bladder filling and gastric digestion had an effect on participants’ heart rate (Fagius and Karhuvaara, 1989; Rossi et al., 1998). The participants completed the recording session in a comfortable chair that was located in a dimly lit room. The heart rate recording was performed with a mobile heart rate monitor (RS 800, Polar Electro Oy; Kempele, Finland) that has been shown to record heartbeats as accurate as mobile electrocardiogram systems (Weippert et al., 2010).

### Heartbeat Detection

Participants’ heartbeat detection was determined during the first part of the recording session. Following an established procedure (Lischke et al., 2020a, b), we asked the participants to count their heartbeats during 25, 35, and 45 s lasting time intervals. They were not informed about the length of the time intervals and they were not allowed to use any measure that may have facilitated the heartbeat detection. We used the number of counted and recorded heartbeats to compute two different indices of participants’ heartbeat detection, a traditional heartbeat detection index^1^ (Schandry, 1981) and an alternative heartbeat detection index^2^ (Hart et al., 2013) that has been shown to be to be less sensitive against outliers than the traditional heartbeat detection index (Garfinkel et al., 2015). We used both heartbeat detection indices in our analyses to test the robustness of our findings.

### Heart Rate Variability

Participants’ vagally mediated heart rate variability was determined during the second part of the recording session. Following an established procedure (Lischke et al., 2018b, 2019), we asked the participants to sit still and to stay awake during a 300 s lasting time interval. The heartbeats that were recorded during this time interval were analyzed with Kubios HRV 2.2 (Tarvainen et al., 2014). The analysis followed the guidelines of the Task Force of the European Society of Cardiology (1996): The recordings were detrended (smoothn priors: λ = 500), visually inspected and artifact corrected (adaptive filtering: cubic spline interpolation) before they were subjected to a time-domain and spectral analysis. The time-domain analysis was used for the determination of a heart rate index (meanHR) and for the determination of two vagally mediated heartrate variability indices (the root mean square of successive differences between consecutive heartbeats, RMSSD; the number of successive heartbeat interval pairs that differ more than 50 ms divided by the total number of all heartbeat intervals, pNN50). The values of these indices were in the range of values that have been reported in comparable samples of participants (Dantas et al., 2018). The spectral analysis was used to determine a respiration index (Thayer et al., 2002), the peak frequency of the high frequency band (pHF). We used the respiration index to adjust the vagally mediated heartrate variability indices for respiration-induced alterations (Weippert et al., 2015). Using both vagally mediated heartrate variability indices in our analyses allowed us to test the robustness of our findings.

### Statistical Analyses

We performed all analyses with the bootstrapping module of SPSS 27 (SPSS Inc., Chicago, IL, United States). Preliminary analyses revealed that the datasets of four participants were incomplete or invalid due to a recording error. We, thus, used the datasets of the remaining 109 participants for the main analyses. The main analyses comprised regression and correlation analyses. Multiple regressions were run to analyze the association between participants’ vagally mediated heart rate variability and heartbeat detection. The vagally mediated heart rate variability indices constituted the predictor variables and the heartbeat detection indices constituted the criterion variables. Participant characteristics that may distort the association between vagally mediated heartrate variability and heartbeat detection were used as additional predictor variables (age, sex, body mass index, physical activity, respiratory activity, smoking status, and medication status). The regression analyses were complemented by correlation analyses. Partial correlations were run to quantify the association between participants’ vagally mediated heart rate variability and heartbeat detection. The vagally mediated heart rate variability indices constituted the dependent variable, the heartbeat detection indices the independent variables and the aforementioned participant characteristics the control variables. We set the significance level for all analyses at α ≤ 0.05 and determined significance values (*p*), effect size measures (*r*, *R*^2^, Δ*R*^2^, and *B*) and 95% confidence intervals (CIs).

## Results

### Associations Between the Vagally Mediated Heart Rate Variability Indices (RMSSD, pNN50) and the Traditional Heartbeat Detection Index (HBD_*SC*_)

**FIGURE 1:**
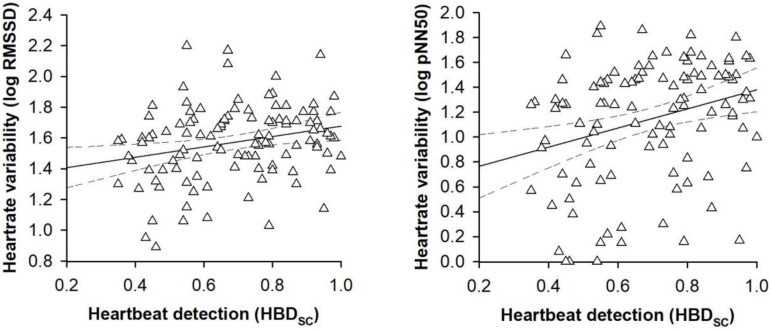
Scatterplots with lines of best fit and 95% confidence intervals demonstrating raw associations between the vagally mediated heart rate variability indices (RMSSD and pNN50) and the traditional heartbeat detection index (HBD_*SC*_).

In the first set of regression models, the traditional heartbeat detection index constituted the criterion variable. Entering the participant characteristics as predictor variables in a first step into the regression models accounted for a significant proportion of the variance in the traditional heartbeat detection index [RMSSD: *R*^2^ = 0.15, *F*(8,100) = 2.13, *p* = 0.040; pNN50: *R*^2^ = 0.15, *F*(8,100) = 2.13, *p* = 0.040, see Table 2]. However, sex was the only predictor variable that turned out be significant in the regression models [RMSSD: *B* = 0.14, *SE B* = 0.05, 95% CI [0.03, 0.23], *t*(100) = 2.37, *p* = 0.015; pNN50: *B* = 0.13, *SE B* = 0.05, 95% CI [0.04, 0.23], *t*(100) = 2.37, *p* = 0.018; see Table 2]. Entering the vagally mediated heart rate variability indices as predictor variables in a second step into the regression models also accounted for a significant proportion of the variance in the traditional heartbeat detection index [RMSSD: Δ*R*^2^ = 0.04, Δ*F*(1,99) = 4.46, *p* = 0.037; pNN50: Δ*R*^2^ = 0.05, *F*(1,99) = 6.13, *p* = 0.015, see Table 2]. The vagally mediated heart rate variability indices were, besides sex [RMSSD: *B* = 0.13, *SE B* = 0.05, 95% CI [0.03, 0.23], *t*(99) = 2.33, *p* = 0.013; pNN50: *B* = 0.12, *SE B* = 0.05, 95% CI [0.02, 0.22], *t*(99) = 2.27, *p* = 0.020; see Table 2], the only significant predictors in the regression models [RMSSD: *B* = 0.15, *SE B* = 0.06, 95% CI [0.02, 0.28], *t*(99) = 2.11, *p* = 0.024; pNN50: *B* = 0.09, *SE B* = 0.04, 95% CI [0.02, 0.16], *t*(99) = 2.48, *p* = 0.009; see Table 2]. Taken together, the regression models suggested that there was a small to medium sized association between the vagally mediated heart rate variability indices and the traditional heartbeat detection index [RMSSD: *r*(99) = 0.21, 95% CI [0.05, 0.37], *p* = 0.037; pNN50: *r*(99) = 24, 95% CI [0.05, 0.41], *p* = 0.015; see Figure 1].

**TABLE 2:** Associations between the vagally mediated heart rate variability indices (RMSSD and pNN50) and the traditional heartbeat detection index (HBD_*SC*_).

	Heartbeat detection (HBD_*SC*_)						Heartbeat detection (HBD_*SC*_)				
Model One	*B*	*SE B*	95% CI	*t*	*p*	Model two	*B*	*SE B*	95% CI	*t*	*p*
*Step one*						*Step one*					
Sex	0.13	0.05	[0.03, 0.23]	2.37	0.015*	Sex	0.13	0.05	[0.04, 0.23]	2.37	0.018*
Age	0.00	0.01	[−0.01, 0.01]	–0.09	0.943	Age	0.00	0.01	[−0.01, 0.01]	–0.09	0.915
Tobacco use	–0.03	0.04	[−0.11, 0.06]	–0.64	0.552	Tobacco use	–0.03	0.04	[−0.11, 0.06]	–0.64	0.545
Medication use	0.04	0.08	[−0.10, 0.21]	0.70	0.610	Medication use	0.04	0.08	[−0.10, 0.20]	0.70	0.574
Contraceptive use	0.03	0.05	[−0.08, 0.14]	0.54	0.545	Contraceptive use	0.03	0.05	[−0.07, 0.14]	0.54	0.559
Body mass index	–0.01	0.01	[−0.02, 0.01]	–0.97	0.342	Body mass index	–0.01	0.01	[−0.02, 0.01]	–0.97	0.312
Physical activity	0.01	0.01	[0.00, 0.02]	1.79	0.066	Physical activity	0.01	0.01	[0.00, 0.02]	1.79	0.068
Respiratory activity (log pHF)	–0.03	0.16	[−0.32, 0.30]	–0.15	0.891	Respiratory activity (log pHF)	–0.03	0.16	[−0.35, 0.30]	–0.15	0.870
*Step two*						*Step two*					
Sex	0.13	0.05	[0.03, 0.23]	2.33	0.013**	Sex	0.12	0.05	[0.03, 0.22]	2.27	0.020*
Age	0.00	0.01	[−0.01, 0.01]	0.00	1.000	Age	0.00	0.00	[−0.01, 0.01]	–0.07	0.946
Tobacco use	–0.02	0.04	[−0.10, 0.07]	–0.44	0.661	Tobacco use	–0.02	0.04	[−0.1, 0.07]	–0.41	0.694
Medication use	0.04	0.08	[−0.1, 0.20]	0.66	0.610	Medication use	0.04	0.07	[−0.08, 0.19]	0.72	0.551
Contraceptive use	0.05	0.05	[−0.06, 0.15]	0.81	0.374	Contraceptive use	0.04	0.05	[−0.06, 0.15]	0.69	0.452
Body mass index	0.00	0.01	[−0.02, 0.01]	–0.50	0.669	Body mass index	0.00	0.01	[−0.02, 0.01]	–0.41	0.687
Physical activity	0.01	0.00	[0.00, 0.02]	1.41	0.120	Physical activity	0.01	0.01	[0.00, 0.02]	1.24	0.185
Respiratory activity (log pHF)	–0.11	0.16	[−0.40, 0.22]	–0.59	0.486	Respiratory activity (log pHF)	–0.10	0.16	[−0.41, 0.25]	–0.53	0.558
Heart rate variability (log RMSSD)	0.15	0.06	[0.02, 0.28]	2.11	0.024*	Heart rate variability (log pNN50)	0.09	0.04	[0.02, 0.16]	2.48	0.014**

Model one: step one: R^2^ = 0.15, F(8,100) = 2.13, p = 0.040*, step two: ΔR^2^ = 0.04, ΔF(1,99) = 4.46, p = 0.037*; model two: step one: R^2^ = 0.15, F(8,100) = 2.13, p = 0.040*, step two: ΔR^2^ = 0.05, ΔF(1,99) = 6.13, p = 0.015*. HBD_SC_, Heartbeat detection – traditional index (Schandry, 1981); pHF, peak of high frequency band (Shaffer and Ginsberg, 2017); RMSSD, root mean square of successive differences between consecutive heartbeats (Shaffer and Ginsberg, 2017); pNN50, number of successive heartbeat interval pairs that differ more than 50 ms divided by the total number of all heartbeat intervals (Shaffer and Ginsberg, 2017). *p ≤ 0.05, **p ≤ 0.01, ***p ≤ 0.001.

### Associations Between the Vagally Mediated Heart Rate Variability Indices (RMSSD, pNN50) and the Alternative Heartbeat Detection Index (HBD_*GA*_)

**FIGURE 2:**
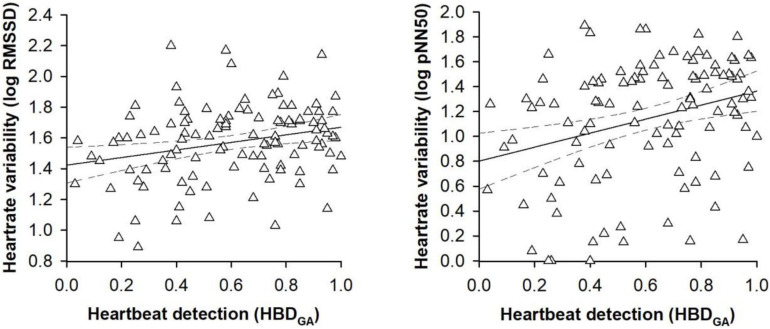
Scatterplots with lines of best fit and 95% confidence intervals demonstrating raw associations between the vagally mediated heart rate variability indices (RMSSD and pNN50) and the alternative heartbeat detection index (HBD_*GA*_).

In the second set of regression models, the alternative heartbeat detection index constituted the criterion variable. Entering the participant characteristics as predictor variables in a first step into the regression models accounted for a significant proportion of the variance in the alternative heartbeat detection index [RMSSD: *R*^2^ = 0.14, *F*(8,100) = 2.12, *p* = 0.041; pNN50: *R*^2^ = 0.14, *F*(8,100) = 2.12, *p* = 0.041; see Table 3]. However, sex was the only predictor variable that turned out be significant in the regression models [RMSSD: *B* = 0.18, *SE B* = 0.08, 95% CI [0.03, 0.23], *t*(100) = 2.36, *p* = 0.017; pNN50: *B* = 0.18, *SE B* = 0.07, 95% CI [0.04, 0.33], *t*(100) = 2.36, *p* = 0.009; see Table 3]. Entering the vagally mediated heart rate variability indices as predictor variables in a second step into the regression models also accounted for a significant proportion of the variance in the alternative heartbeat detection index [RMSSD: Δ*R*^2^ = 0.04, Δ*F*(1,99) = 4.79, *p* = 0.031; pNN50: Δ*R*^2^ = 0.05, *F*(1,99) = 6.70, *p* = 0.011; see Table 3]. The vagally mediated heart rate variability indices were, besides sex [RMSSD: *B* = 0.18, *SE B* = 0.08, 95% CI [0.03, 0.34], *t*(99) = 2.32, *p* = 0.022; pNN50: *B* = 0.17, *SE B* = 0.07, 95% CI [0.04, 0.32], *t*(99) = 2.25, *p* = 0.015; see Table 3], the only significant predictors in the regression models [RMSSD: *B* = 0.23, *SE B* = 0.09, 95% CI [0.04, 0.40], *t*(99) = 2.19, *p* = 0.020; pNN50: *B* = 0.13, *SE B* = 0.05, 95% CI [0.03, 0.22], *t*(100) = 2.59, *p* = 0.007; see Table 3]. Taken together, the regression models suggested that there was a small to medium sized association between the vagally mediated heart rate variability indices and the alternative heartbeat detection index [RMSSD: *r*(99) = 0.21, 95% CI [0.05, 0.37], *p* = 0.031; pNN50: *r*(99) = 0.25, 95% CI [0.06, 0.42], *p* = 0.011; see Figure 2].

**TABLE 3:** Associations between the vagally mediated heart rate variability indices (RMSSD and pNN50) and the alternative heartbeat detection index (HBD_*GA*_).

	Heartbeat detection (HBD_*GA*_)						Heartbeat detection (HBD_*GA*_)				
Model One	*B*	*SE B*	95% CI	*t*	*p*	Model two	*B*	*SE B*	95% CI	*t*	*p*
*Step one*						*Step one*					
Sex	0.18	0.08	[0.03, 0.34]	2.36	0.017*	Sex	0.18	0.07	[0.04, 0.33]	2.36	0.009**
Age	0.00	0.01	[−0.02, 0.01]	–0.49	0.668	Age	0.00	0.01	[−0.02, 0.01]	–0.49	0.656
Tobacco use	–0.03	0.06	[−0.14, 0.09]	–0.45	0.690	Tobacco use	–0.03	0.06	[−0.13, 0.09]	–0.45	0.696
Medication use	0.06	0.11	[−0.15, 0.29]	0.66	0.555	Medication use	0.06	0.11	[−0.14, 0.28]	0.66	0.583
Contraceptive use	0.04	0.08	[−0.12, 0.18]	0.53	0.579	Contraceptive use	0.04	0.08	[−0.1, 0.20]	0.53	0.565
Body mass index	–0.01	0.01	[−0.03, 0.01]	–1.05	0.290	Body mass index	–0.01	0.01	[−0.03, 0.01]	–1.05	0.318
Physical activity	0.01	0.01	[0.00, 0.03]	1.86	0.063	Physical activity	0.01	0.01	[0.00, 0.03]	1.86	0.064
Respiratory activity (log pHF)	0.02	0.23	[−0.42, 0.49]	0.07	0.930	Respiratory activity (log pHF)	0.02	0.23	[−0.42, 0.45]	0.07	0.933
*Step two*						*Step two*					
Sex	0.18	0.08	[0.03, 0.34]	2.32	0.022*	Sex	0.17	0.07	[0.04, 0.32]	2.25	0.015*
Age	0.00	0.01	[−0.02, 0.01]	–0.40	0.726	Age	0.00	0.01	[−0.02, 0.01]	–0.47	0.688
Tobacco use	–0.01	0.06	[−0.13, 0.10]	–0.23	0.825	Tobacco use	–0.01	0.06	[−0.12, 0.1]	–0.20	0.842
Medication use	0.05	0.10	[−0.15, 0.27]	0.61	0.568	Medication use	0.06	0.10	[−0.13, 0.27]	0.68	0.552
Contraceptive use	0.07	0.08	[−0.09, 0.21]	0.82	0.384	Contraceptive use	0.06	0.08	[−0.09, 0.2]	0.70	0.468
Body mass index	–0.01	0.01	[−0.03, 0.01]	–0.56	0.553	Body mass index	0.00	0.01	[−0.03, 0.02]	–0.47	0.665
Physical activity	0.01	0.01	[0.00, 0.03]	1.47	0.123	Physical activity	0.01	0.01	[0.00, 0.02]	1.29	0.162
Respiratory activity (log pHF)	–0.10	0.24	[−0.57, 0.4]	–0.40	0.647	Respiratory activity (log pHF)	–0.08	0.23	[−0.54, 0.35]	–0.32	0.734
Heart rate variability (log RMSSD)	0.23	0.09	[0.04, 0.4]	2.19	0.020*	Heart rate variability (log pNN50)	0.13	0.05	[0.03, 0.22]	2.59	0.007**

Model one: step one: R^2^ = 0.14, F(8,100) = 2.12, p = 0.031*, step two: ΔR^2^ = 0.04, ΔF(1,99) = 4.79, p = 0.031*; model two: step one: R^2^ = 0.14, F(8,101) = 2.12, p = 0.041*, step two: ΔR^2^ = 0.05, ΔF(1,99) = 6.70, p = 0.011*. HBD_GA_, Heartbeat detection – alternative index (Garfinkel et al., 2015); pHF, peak of high frequency band (Shaffer and Ginsberg, 2017); RMSSD, root mean square of successive differences between consecutive heartbeats (Shaffer and Ginsberg, 2017); pNN50, number of successive heartbeat interval pairs that differ more than 50 ms divided by the total number of all heartbeat intervals (Shaffer and Ginsberg, 2017). *p < 0.05, **p < 0.01, ***p < 0.001.

## Discussion

To explore the possibility that our interoceptive accuracy depends on our ability to engage prefrontal and (para-)limbic brain regions for this matter (Critchley et al., 2004; Pollatos et al., 2005, 2007a; Kuehn et al., 2016), we administered cardiac measures of interoceptive accuracy and prefontal-(para-)limbic engagement to a sample of young adults. Interoceptive accuracy was measured with a heartbeat detection task and prefrontal-(para-)limbic engagement with a heart rate recording. We used the heartbeat detection task to determine two different heartbeat detection indices and the heart rate recording to determine two different vagally mediated heart rate variability indices (Shaffer and Ginsberg, 2017). The vagally mediated heart rate indices were both positively associated with the heartbeat detection indices, regardless whether the traditional or alternative heartbeat detection index were considered in the regression and correlation analyses. The regression and correlation analyses were well-powered and well-controlled, ruling out that the association between the vagally mediated heart rate variability index and the heartbeat detection indices was spurious. We, thus, found the expected association between vagally mediated heart rate variability and heartbeat detection. The association between vagally mediated heart rate variability and heartbeat detection supports the idea that our interoceptive accuracy depends on our ability to engage prefrontal and (para-)limbic brain regions for the perception and interpretation of cardiac changes.

To understand the association between vagally mediated heart rate variability and heartbeat detection, we have to delineate the processes that determine the performance on the heartbeat detection task. Heartbeat detection relies on executive control processes (Critchley and Garfinkel, 2017), in particular on those that are related to attention. For an accurate perception and interpretation of cardiac changes, attention has to be shifted from the outside to the inside of the body, to be shielded against sensations from the outside of the body and to be focused on sensations inside the body. Attention shifting, attention shielding and attention focusing are executive control processes that are driven by activity changes in prefrontal brain and (para-)limbic regions (Petersen and Posner, 2012). Activity changes in prefrontal and (para-)limbic brain regions are associated with vagally mediated heart rate variability (Thayer et al., 2012; Ruiz Vargas et al., 2016), indicating that vagally mediated heart rate variability may also be associated with these executive control processes. Vagally mediated heart rate variability is, in fact, associated with executive control processes (Zahn et al., 2016; Holzman and Bridgett, 2017), including attention shifting, attention shielding and attention focusing (Hansen et al., 2003; Williams et al., 2016; Siennicka et al., 2019; Sorensen et al., 2019). The association between vagally mediated heart rate variability and heartbeat detection may, thus, be mediated by executive control processes that are driven by activity changes in prefrontal and (para-)limbic brain regions.

There are several prefrontal and (para-)limbic brain regions that may mediate the association between vagally mediated heart rate variability and heartbeat detection through executive control processes. These brain regions are organized in networks that are implicated in the up- and downregulation of cardiac changes (Thayer and Lane, 2009), the perception and interpretation of cardiac changes (Schulz, 2016) and the execution of externally and internally oriented attention changes (Petersen and Posner, 2012). Some brain regions are part of more than one network. These brain regions provide functional and structural connections between the networks (Bullmore and Sporns, 2012). Most connections are provided by the anterior cingulate cortex and the insula (Medford and Critchley, 2010). These connections make the anterior cingulate cortex and the insula to central network hubs that coordinate the interplay between the networks (Dosenbach et al., 2007; Sridharan et al., 2008). The anterior cingulate cortex and the insula monitor and regulate the activity of all brain regions in the networks (Barrett and Simmons, 2015), which explains why activity changes in the anterior cingulate cortex and insula are closely associated with vagally mediated heart rate variability (Chang et al., 2013; Allen et al., 2015; Jennings et al., 2016), heartbeat detection (Critchley et al., 2004; Pollatos et al., 2005; Kuehn et al., 2016) and attention (Seeley et al., 2007; Eckert et al., 2009; Shulman et al., 2009). Activity changes in the anterior cingulate cortex and the insula may, thus, trigger executive control processes that mediate the association between vagally mediated heart rate variability and heartbeat detection.

To illustrate the importance of the anterior cingulate cortex and the insula for mediating the association between vagally mediated heart rate variability and heartbeat detection through executive control processes, we only have to take a look at some of the most common mental disorders (Paulus and Stein, 2010). Mood and anxiety disorders are characterized by alterations in vagally mediated heart rate variability (Licht et al., 2008, 2009), heartbeat detection (Pollatos et al., 2009; Dunn et al., 2010) and executive control processes (Mogg et al., 1992, 1995; Lim and Kim, 2005). The alterations in vagally mediated heart rate variability and heartbeat detection are related to alterations in anterior cingulate cortex and insula activity during the perception and interpretation of cardiac changes (Caseras et al., 2013; Avery et al., 2014; Wiebking et al., 2015; Cui et al., 2020; DeVille et al., 2020), presumably via alterations in executive control processes (Mitterschiffthaler et al., 2008; Etkin et al., 2010). The alterations in anterior cingulate cortex and insula activity account for severe alterations in emotion, cognition and behavior (Caseras et al., 2013; Avery et al., 2014; Wiebking et al., 2015; Cui et al., 2020; DeVille et al., 2020), indicating that interoceptive deficits play an important role in the etiology and pathogenesis of mood and anxiety disorders (Paulus and Stein, 2010).

Considering the importance of interoceptive deficits for the etiology and pathogenesis of mood and anxiety disorders (Paulus and Stein, 2010), we need measures that allow us to identify those of us whose interoceptive deficits put them at risk for these disorders. As we have shown, cardiac measures may be useful for this purpose. We combined a cardiac measure of interoceptive accuracy, the heartbeat detection index, with a cardiac measure of prefrontal-(para-)limbic engagement, the vagally mediated heart rate variability index. Combining these measures allowed us to demonstrate that our interoceptive accuracy depends on our prefrontal and (para-)limbic engagement during the perception and interpretation of cardiac changes. It should be noted, however, that we only employed cardiac but not neural measures in our investigation. We, thus, can only assume that vagally mediated heart rate variability reflected prefrontal-(para-)limbic engagement during the perception and interpretation of cardiac changes. Future investigations that supplement cardiac measures with neural measures may help to test this assumption with more rigor (e.g., measuring vagally mediated heart rate variability and heartbeat detection during functional or structural imaging). Future investigations should also employ a more rigorous control of participant characteristics that affect cardiac measures than we did in our investigation (e.g., excluding participants with medication use or caffeine use). We hope that our investigation opens an avenue for these types of investigations because we believe that cardiac measures are a promising tool for researchers in the field of interoception. Cardiac measures can be obtained from unobtrusive heart rate recordings that do not require dedicated staff or equipment. These measures may, thus, be interesting for researchers who need to investigate the association between interoception and prefrontal-(para-)limbic engagement in a time- and cost-efficient manner.

## Data Availability Statement

The datasets presented in this article are not readily available because of ethical restrictions. Requests to access the datasets should be directed to AL, alexander.lischke@uni-greifswald.

## Ethics Statement

This study was reviewed and approved by the Ethics Committee of the University of Rostock. The participants provided their written informed consent to participate in the study.

## Author Contributions

AL, RP, and MW designed the study. AM-M and MW collected the data. AL and MW analyzed the data. AL and RP wrote the manuscript. AM-M, MW, and RP contributed to writing, reviewing, and editing of the manuscript. All authors approved the final version of the manuscript.

## Conflict of Interest

The authors declare that the research was conducted in the absence of any commercial or financial relationships that could be construed as a potential conflict of interest.

## Footnotes

1 $1{\text{HBD}}_{\text{SC}}=\frac{1}{3}\sum \left(1-\frac{\left(\left|n{\text{heartbeats}}_{\text{real}}-n{\text{heartbeats}}_{\text{counted}}\right|\right)}{n{\text{heartbeats}}_{\text{real}}}\right)$

2 $2{\text{HBD}}_{\text{GF}}=\frac{1}{3}\sum \left(1-\frac{\left(\left|n{\text{heartbeats}}_{\text{real}}-n{\text{heartbeats}}_{\text{counted}}\right|\right)}{\left(n{\text{heartbeats}}_{\text{real}}+n{\text{heartbeats}}_{\text{counted}}\right)/2}\right)$
